# Protein kinase B/AKT isoform 2 drives migration of human mesenchymal stem cells

**DOI:** 10.3892/ijo.2012.1700

**Published:** 2012-11-15

**Authors:** ZRINKA BULJ, SERENA DUCHI, ALESSANDRO BEVILACQUA, ALESSANDRO GHERARDI, BARBARA DOZZA, FILIPPO PICCININI, GIULIA ADALGISA MARIANI, ENRICO LUCARELLI, SANDRO GIANNINI, DAVIDE DONATI, SANDRA MARMIROLI

**Affiliations:** 1 Cellular Signalling Laboratory, Department of Biomedical Sciences, 40126 Bologna;; 2 Osteoarticular Regeneration Laboratory, Rizzoli Orthopaedic Institute, 40136 Bologna;; 3 Advanced Research Center on Electronic Systems for Information and Communication Technologies ‘E. De Castro’ (ARCES), 40125 Bologna;; 4 Department of Electronics, Computer Science and Systems (DEIS), 40136 Bologna;; 5 Department of Biomedical Sciences, 40126 Bologna;; 6 Cellular Signalling Laboratory, Department of Surgery, Medicine, Dentistry and Morphological Sciences, 41124 Modena, Italy

**Keywords:** mesenchymal stem cells, phosphoinositide-3 kinase/protein kinase B pathway, cell migration, protein kinase B Inhibitors, wound healing

## Abstract

This study was designed to investigate the migratory behavior of adult human mesenchymal stem cells (MSC) and the underlying mechanism. Cell migration was assessed by transwell, wound healing and time-lapse *in vivo* motility assays. Pharmacological inhibitors were used to determine the potential mechanism responsible for cell migration and invasion. The tests that were implemented revealed that MSC were fairly migratory. Protein kinase B (AKT) was strongly activated at the basal level. Through our analyses we demonstrated that pharmacological inactivation of AKT2 but not AKT1 significantly decreased cell migration and invasion. Although preliminary, collectively our results indicate that AKT2 activation plays a critical role in enabling MSC migration.

## Introduction

Mesenchymal stem cells (MSC) are a rare population of bone marrow-derived non-hematopoietic fibroblast-like cells, representing ∼0.001–0.01% of the nucleated cells in the marrow. The accessibility, ease of handling, and enormous expansion potential of MSC, together with their capacity for self-renewal (reviewed in ref. 1) and amenability to allogeneic transplantation (2), provide a nearly unlimited supply of cells for therapeutic applications. MSC are attractive candidates for manipulation as they can easily be isolated from patients, cultured *in vitro*, and autologously transplanted into patients, thus overcoming the difficulties related to immune rejection of transplanted cells (3). A significant improvement in understanding MSC biology in recent years has paved the way to their potential clinical use. In particular, MSC are emerging as novel cell-based delivery agents. Indeed, thanks to inherent tumor-trophic migratory properties, MSC could vehicle effective targeted therapy to isolated tumors and metastatic disease. However, defective migration is a major caveat in cell-based therapy as systemic stem cell transplantation requires efficient cell homing, and is therefore of utmost importance to understand the underlying molecular mechanisms driving MSC movement to specific target sites (reviewed in ref. 4).

Phosphoinositide-3 kinase (PI3K)/AKT signaling circuit is a well established regulator of important functions in cells, such as cell cycling, survival and cell growth (5). In addition, recent studies have demonstrated that AKT plays a pivotal role in cell migration and invasion (6–8). In particular, AKT enhances actin remodeling by a number of possibly interconnected mechanisms, in several cell systems. AKT modulates actin bundling by direct actin binding, and generates membrane protrusions through downstream activation of Rac1 and Cdc42 (9,10). Besides, PI3K/AKT signals induce also the remodeling of actin through the activation of p70S6K, leading to cell migration and cell invasion (11). Furthermore, AKT promotes actin organization and cell motility mediated by a number of substrates and/or interactors, such as Girdin/AKT-phosphorylation enhancer (APE) (12), the actin bundling protein Palladin (13) and the mechano-protein and AKT-substrate ANKRD2 (14). Accordingly, PI3K/AKT signals are considered to be crucial in cell migration by controlling actin dynamics.

Remarkably, it has been demonstrated that AKT isoform 1 and AKT isoform 2 exert opposing functions in breast cancer cell migration and metastasis (6,7,13,15). There is little information, however, on their role in other cell models. We therefore aimed to elucidate the contribution of AKT to MSC migration by specific pharmacological inhibition.

## Materials and methods

### Mesenchymal stem cell culture

Bone marrow (BM) samples were obtained from patients undergoing surgery at Rizzoli Orthopaedic Institute after obtaining informed consent. Bone marrow derived MSC cultures were obtained as previously described (16). Cells were transferred to 150-cm^2^ culture flasks with α-Modified minimum Essential medium (α-MEM; BioWhittaker, Lonza, Verviers, Belgium) supplemented with 20% lot-selected fetal bovine serum (FBS; Gibco, Invitrogen, Paisley, UK) and GlutaMAX™ 1% (Invitrogen), incubated in a humidified atmosphere at 37°C with 5% CO_2_ and medium changed every 3–4 days. When the cells reached approximately 70–80% confluence, they were detached by mild trypsinization (TripLe™ Select, Invitrogen) for 5 min at 37°C and counted. Then, 1/3 of them were reseeded into a new 150 cm^2^ flask.

MSC were recognized by their ability to proliferate in culture with adherent, spindle-shape morphology. The total number of cells obtained at each passage was extrapolated from the counted representative samples; the number was calculated by multiplying the number of cells counted by the number of flasks available at each passage. Cell number and cell viability were assessed for each passage by NucleoCounter^®^ (ChemoMetec, Lillerød, Denmark), that detects non-viable cells by using propidium iodide nuclear staining, and determines cell viability by calculating the ratio of total and non-viable cell number. Further characterization of MSC was performed by cytofluorimetric analysis of cell surface markers at passage two. MSC resulted positive for CD44, CD73, CD90, CD105, CD146 and negative for CD34 and CD45 (17). The multilineage potential was evaluated by differentiation assays as previously described (18). After the appropriate stimulations, cells positively differentiated in osteogenic, adipogenic and condrogenic lineages.

### Drug treatments

Cells were seeded 1×10^4^ cells/cm^2^ density in complete medium and starved overnight with 0.2% FBS medium. On the third day, culture was treated for 30 min with PI3K Inhibitor LY-294002 (#L9908, Sigma-Aldrich, St. Louis, MO, USA) at 10 *μ*M and AKT Inhibitor IV (#B2311, Sigma-Aldrich) at 2.5 *μ*M concentration. Inhibitor VIII (#202048, Santa Cruz Biotechnology, Santa Cruz, CA, USA) was used at 1 *μ*M to inhibit AKT1/2 and Inhibitor XII (#124029, Calbiochem, La Jolla, CA, USA) was used at 5 *μ*M for AKT2 isoform. Specific Inhibitors for singular isoforms were used for 1 h.

### Immunofluorescence microscopy

Cells were seeded (2.5×10^4^) onto glass coverslips and fixed for 10 min in 4% formaldehyde at RT, as described (19). After three washes in PBS, cells were permeabilized for 10 min in PBT (PBS + 0,1% Triton X-100), then incubated in blocking solution (1X PBS + 5% BSA) for 30 min. Primary antibody anti-Vinculin (1:100, #MAB3574, Chemicon, Temecula, CA, USA) diluted in blocking solution was added and incubated for 1 h at RT. After three washes in 1X PBS, secondary antibody anti-mouse Cy3 (1:100, #C2181, Sigma-Aldrich) was added diluted in 1X PBS and incubated for 45 min at RT. Cortical actin was stained using FITC-Phalloidin (1:500, #P5282, Sigma-Aldrich) and added directly during secondary antibody incubation. Nuclei were stained by incubating cells with Hoechst 33342 (1:2000, #H1399, Invitrogen) for 10 min at RT (20). Coverslips were mounted after washes in PBS in Fluoromount-G (Southern Biotech, Birmingham, AL, USA) solution and analyzed with a NikonTiE microscope equipped with the fully automated A1 confocal laser that incorporates the resonant scanner with a resonance frequency of 7.8 kHz that allows high-speed imaging (A1R), and equipped with DS-QiMc-U2 12 bit camera. Digital images were processed using Matlab and ImageJ software for the analysis and Photoshop software for visualization purposes without biased manipulations (21).

### Preparation of cell extracts and electrophoresis

Cell extracts were obtained as previously reported (22). Briefly, sub-confluent cells were directly extracted by addition of RIPA buffer (20 mM Tris-Cl, pH 7.0, 1% NP-40, 150 mM NaCl, 10% glycerol, 10 mM EDTA, 20 mM NaF, 5 mM sodium pyrophosphate, 1 mM Na_3_VO_4_) and freshly added Sigma-Aldrich Protease Inhibitor Cocktail at 4°C for 20 min. Lysates were cleared by centrifugation. Equal amounts of lysates were loaded on 7,5% Bis-Tris-SDS-polyacrylamide gels (Bio-Rad, Hercules, CA, USA), transferred to Immobilon-P membranes (Millipore Corporation, Billerica, MA, USA), probed with the indicated antibodies by using SNAP i.d.^®^ 2.0 System (Millipore Corporation) and detected by chemiluminescence with Supersignal kit (Pierce, Rockford, IL, USA).

The primary antibodies used: anti-actin 1:1,000 (#sc-8432), anti-AKT 1:1,000 (#sc-1619), from Santa Cruz Biotechnology and anti-pS473AKT 1:1,000 (#4051) from Cell Signalling Technology (Denvers, MA, USA). Secondary antibodies were anti-mouse (#sc-2031) and anti-rabbit (#2317) HRP-conjugated from Santa Cruz Biotechnology and were used 1:5,000 dilution.

### Scratch wound healing assay

Scratch wound tests were performed in a 6-well plate in confluent culture. Cell suspensions (1,000 *μ*l) containing 3×10^5^ cells in 0.2% FBS medium were added to each well and left overnight to allow cell adhesion. Immediately before starting the assay, the scratch was performed by using 200 *μ*l tips. Closing of the wound was monitored along 24 h with a Nikon Eclipse TE2000-U inverted microscope, and calculated as Migrated Cell Surface Area/Total Surface Area × 100 with a web-based image automated analysis software developed by the Koumoutsakos group (CSE Lab), at ETH Zürich (23).

### Cytotoxicity and cell proliferation assay

Cytotoxicity and cell proliferation were evaluated using AlamarBlue^®^ kit (AbD Serotec, Oxford, UK) according to the manufacturer’s instructions. Briefly, the test was performed in 96-well plate by seeding 3×10^3^/cm^2^ in 0.2% FBS medium and treated or not with inhibitors. The reagent was added to the sample for 4 h incubation, at the end of which the indicator passes from the Redox oxidized form (blue) to the reduced form (red) according to the number of cells that are found in the sample. Absorbance was monitored at 570 nm and 600 nm.

### Cell cycle and apoptosis analysis

For detection and quantification of cell cycle distribution samples containing 5×10^5^ cells were harvested by centrifugation, fixed in cold ethanol and subjected to propidium iodide (PI) staining as previously described (24).

### Migration assay

Migration assays were performed in transwell inserts with 8-*μ*m pore uncoated membrane filters (Corning Incorporated, Corning, NY, USA). Cells were trypsinized and seeded onto the upper chambers, in 0.2% FBS medium (4×10^4^ cells/well in 100 *μ*l). The bottom chambers were filled with 10% FBS medium (600 *μ*l). Cells were allowed to migrate for 6 h at 37°C. Then, the upper side of the filters was carefully washed with cold PBS and non-migrated cells at the top of the filter were removed using a cotton swab. Cells that had migrated to the bottom of the filters were fixed and stained with Hema 3^®^ Protocol Stain (Fisher Diagnostics, Middletown, CT, USA), and imaged with a Nikon Eclipse TE2000-U inverted epifluorescence microscope. Stained cells were counted in 10 pictures taken at x400 magnification. The average of cell number counted per field was multiplied by the ratio between the surfaces (total/counted) to determine the total number of migrated cells. Inhibition of migration was calculated normalizing data to control cells.

### In vivo imaging and motility analysis

Cells were plated at a density of 2.5×10^4^ cells in 35-mm Glass Bottom Dishes (MatTek Corporation, Ashland, OR, USA) and let to adhere overnight. Drug treatments were performed as described above. Images were recorded with a NikonTi Eclipse microscope equipped with a CO_2_ temperature controller atmosphere (Okolab, Ottaviano, NA, Italy) and a DS-QiMc-U2 12 bit camera. A 10X Plan-Apochromatic Ph1 DL 0.25NA objective was used, yielding 0.91 *μ*m/pixel in the acquired images. Total duration of recording is 15 h with a time frame interval of 5 min. Three plates were acquired for each of the control (CTRL) and treated (XII) MSC cultures for a total of six sequences of 180 frames each. Among these, three representative Regions of Interest (ROI) (labeled a,b,c) for each plate were considered for the motility analysis. In order to assess cells motility in treated and control MSC, we employed our tracking algorithm designed for this purpose. Image processing methods were devised to first detect the cell regions around the nuclei on each frame of the considered sequences through non-linear and morphological filters. Then, a combined feature-based and morphology-based tracking method was employed to track each cell center of mass (CM) along the sequence of frames to achieve the cell trajectory. Since the image illumination may change during the time-lapse sequence, phase contrast images could present artifacts that mislead the tracking stage. Also, during the acquisition of the six sequences, the repositioning error of the motorized stage where the plates were placed yielded x/y displacement on the imaging plane, this causing images not to be perfectly aligned. While the latter problem was easily fixed by phase-correlation registration methods, the former needed manual intervention. Trajectories recovered from the sequence of images were therefore analyzed by two experts to select only those with a high confidence, while the others were discarded.

### Statistical analysis

All the images shown in this paper are representative of at least three independent experiments carried out under the same conditions. Statistical analysis were performed by ANOVA test followed by Bonferroni’s multiple comparison test. P<0.05 were considered statistically significant. Data are expressed as the mean ± standard deviation (SD).

## Results

### PI3K/AKT signaling pathway inhibition triggers actin remodeling of MSC

**Figure 1.**
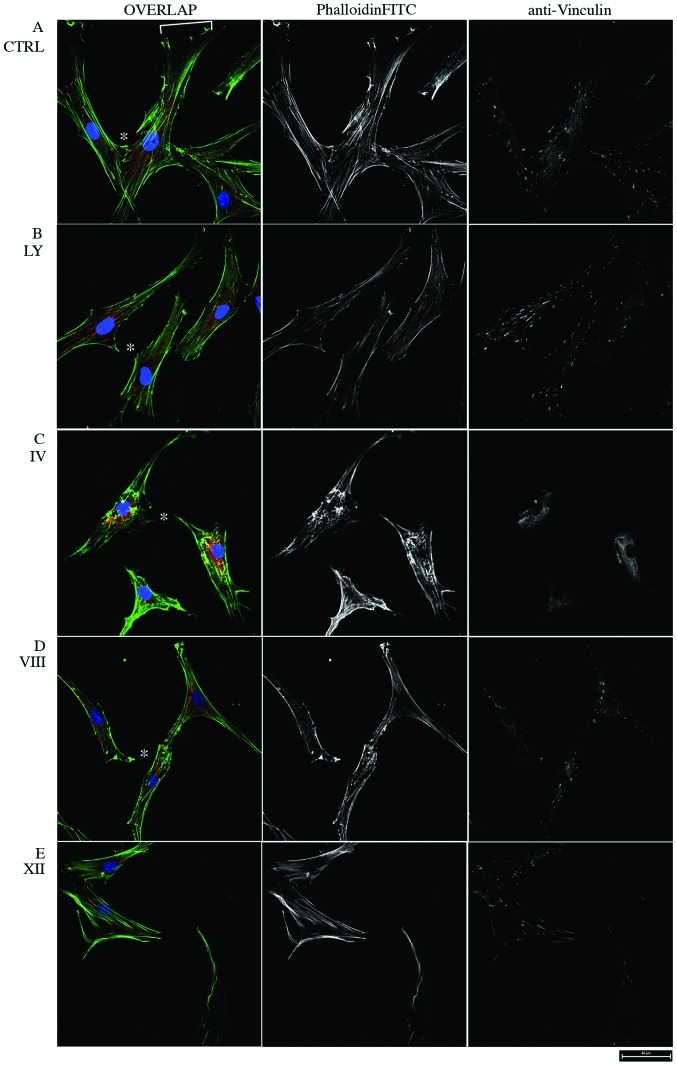
Cytoskeletal remodeling of MSC exposed to PI3K/AKT Inhibitors. (A) Control MSC. (B) MSC treated for 30 min with 10 *μ*M Inhibitor LY294002. (C) MSC treated for 30 min with 2.5 *μ*M AKT Inhibitor IV. (D) MSC treated for 60 min 1 *μ*M with AKT1/2 Inhibitor VIII. (E) MSC treated for 60 min with 5 *μ*M AKT2 Inhibitor XII. Immunofluorescence was performed using anti-vinculin antibody and FITC conjugated phalloidin actin, shown in red and green in Overlap panels, respectively. Hoechst staining is shown in blue in Overlap images. Pictures were taken using a 60X PlanApo VC Oil DIC N2 objective. White bracket in (A) pinpoints the migration front leading edge. Asterisks in B, C and D indicate the points of intercellular contacts loss. Scale bar is 50 *μ*m in all panels. Representative pictures are shown.

MSC were exposed for 30 or 60 min to increasing concentrations of well characterized PI3K/AKT inhibitors (25), in order to identify concentrations that do not compromise cell viability (data not shown), and the morphology was observed by confocal microscopy (Fig. 1). In control MSC, PhalloidinFITC staining showed a dense network of F-actin bundles with tight, parallel stress fibers and adhesion foci and a well shaped migration front leading edge (Fig. 1A). Conversely, MSC treated with the PI3K Inhibitor LY 294002 were characterized by reduced actin bundling and partial loss of cell-cell contacts and cell migration front (Fig. 1B). Moreover, the broad PI3K/Akt Inhibitor IV leds to collapse of actin bundles and complete loss of intercellular and focal adhesions, as visualized by vinculin staining (Fig. 1C). The AKT-dependent actin skeleton remodeling was monitored further in response to PH domain-dependent, non-ATP-competitive and isoform-specific Inhibitors VIII and XII (AKTi VIII and XII), that potently inhibit Akt1/2 and Akt2 respectively, but not other AGC kinases (26), showing that both AKTi VIII and XII triggered partial loss of actin fibers and cell rounding, whereas focal adhesions became restricted to discrete protruding portions of the cell periphery (Fig. 1D and E). Thus, in good agreement with previous reports in other cell types, alteration of cell morphology and actin skeleton organization by pharmacological inhibition of PI3K/AKT indicates a key role of this pathway in the cytoskeletal remodeling of MSC.

**Figure 2.**
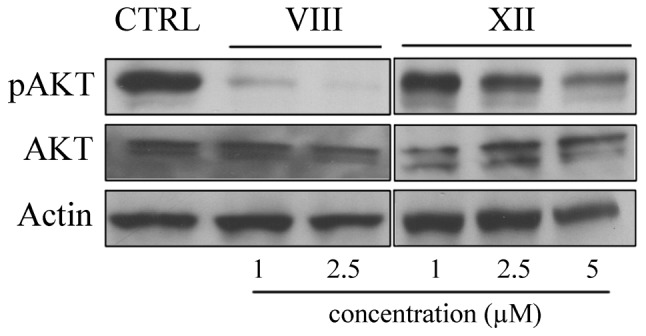
AKT activity is efficiently blocked by specific inhibitors. Immuno-blotting analysis of activated AKT (pAKT) and total AKT (AKT1/2) levels in vehicle and AKTi VIII (1, 2.5 *μ*M) and XII (1, 2.5, 5 *μ*M) 60 min treated MSC. Anti-actin shows equal loading.

As a read-out of treatment efficacy, phosphorylation of Akt S473 was monitored by western blot analysis. As expected, 60 min upon treatment AKT S473 phosphorylation was decreased in a dose-dependent manner by both inhibitors (Fig. 2).

### MSC migration and motility are affected by AKT inhibition

Defective migration is a major limitation in cell-based therapy. Therefore, in light of its well described role in actin remodeling linked to cell migration in several cell models, and based on the above observations, we aimed to investigate deeper AKT functions in MSC. Since wound repair is a homeostatic process in which cell migration features prominently, we first examined the effect of AKT inhibition on the migration of MSC by means of the monolayer scratch wound healing assay.

**Figure 3.**
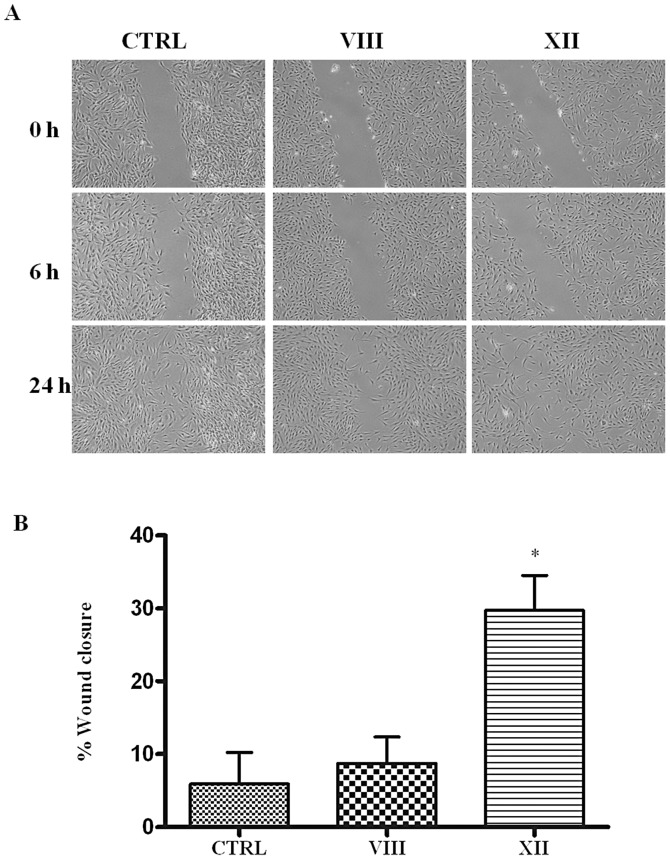
AKT inhibitors affect MSC migration. (A) Phase contrast images of MSC treated with AKTi VIII and XII. (B) Graphical view showing the percentage of wound closure during 24 h. ^*^p<0.05. AKTi XII delays wound closure.

Remarkably, while vehicle-treated MSC were able to close the scratch in 24 h, treatment with the AKTi XII, that selectively inhibits isoform 2, markedly blunted migration and after 24 h the wound closure was still incomplete (Fig. 3). In contrast, treatment with AKTi VIII, that inactivates both isoform 1 and 2, reduced migration to a lesser extent, resulting in an almost complete wound closure after 24 h.

### Cell migration is not influenced by cell proliferation and cytotoxicity

**Figure 4.**
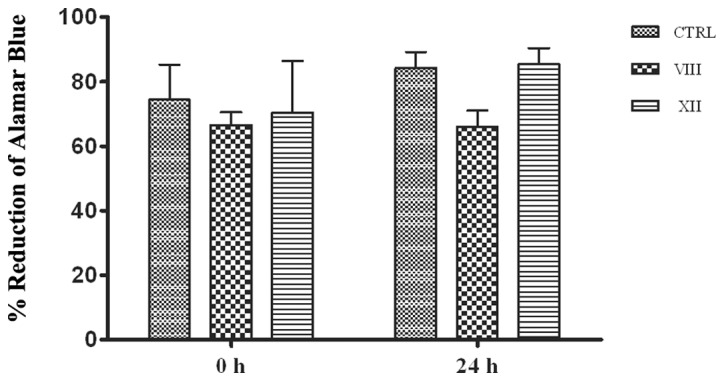
Cytotoxicity and proliferation of MSC upon AKT inhibition. % of Alamar Blue reduction at time zero (0 h) and 24 h after drug-treatment.

Cell proliferation itself can promote wound closure. Besides, AKT inhibitors in turn might affect wound closure by slowing proliferation. To exclude this bias, proliferation of vehicle- or AKTi-treated MSC was calculated by Alamar Blue assay (Fig. 4). The test is based on the ability of metabolically active cells to convert the reagent from a blue to red colorimetric indicator. Damaged and non-viable cells have lower innate metabolic activity and thus generate a proportionally lower signal. Total metabolic activity, checked at time zero (0 h) and after 24 h, indicates no substantial variation following AKT inhibition. Thus, the drugs did not alter cell growth for the duration of the scratch wound test. Furthermore, cell cycle progression measured by flow cytometry confirmed cell viability and showed that cell cycle distribution was comparable between samples (Table I). Moreover, no remarkable apoptosis was detected, excluding cytotoxic effects of the above-mentioned drugs in this context. We conclude that the effects observed on MSC motility were independent of changes in MSC proliferation as well as alteration of metabolic activity.

### MSC migration is compromised by AKT2 isoform inhibition

**Figure 5.**
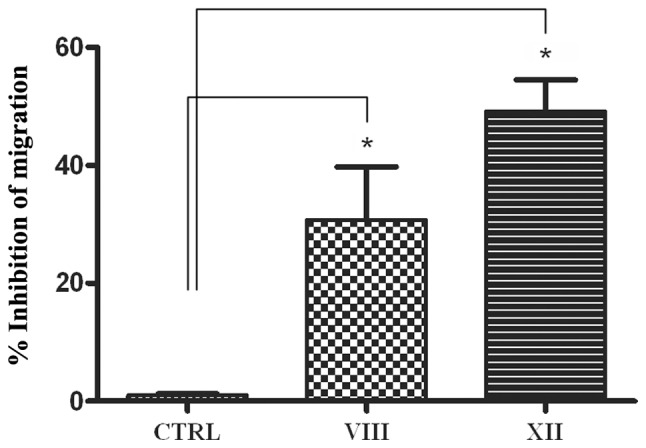
Effect of AKT Inhibitors on MSC migration. MSC migration was examined after AKTi VIII and XII treatment by transwell migration assay. ^*^p<0.05. Error bars indicate SD.

**Figure 6.**
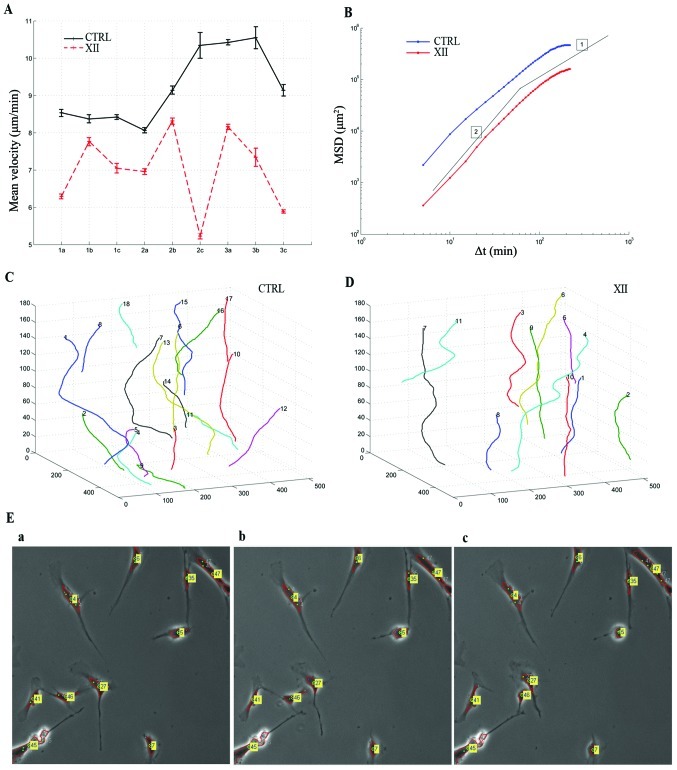
*In vivo* cell tracking analysis. (A) Mean velocity (Y-axis) of cell tracks in 9 different image sequences (X-axis) of control (CTRL) and treated (XII) MSC. Error bar indicates SD. (B) MSD <r^2^(Δt)> versus time lag Δt for CTRL and XII MSC. Thin lines indicate logarithmic slopes of 1 and 2. (C and D) Trajectory plot of tracked cells in the sequence (C) CTRL 3c and (D) XII 3c. XY-plane units are in pixel, whilst Z unit is video frame number. (E) Three subsequent frames (Δt = 5 min) extracted from the sequence CTRL 3c showing tracked cells.

The effect of AKT inhibitors on MSC chemotactic mobility was assessed using 3D transwell migration assays. Consistent with previous reports (27), MSC control cells migrate toward the bottom chamber. Remarkably, treatment with the AKTi XII compound inhibited MSC migration, indicative of a promigratory function of AKT2 in human MSC (Fig. 5). Conversely, combined inhibition of AKT1 and AKT2 by Inhibitor VIII leads to a much weaker effect with respect to inactivation of AKT2 alone. Based on these results, we next tested AKTi XII on 2D-MSC motility through time-lapse *in vivo* microscopy. Fig. 6A shows the plot of measured mean velocity (*μ*m/min) along the cell trajectories for the 18 analyzed sequences. Control cells show a higher velocity than treated cells. The measured mean velocity among the whole sequences is 9.22±0.98 *μ*m/min for the control and 6.99±1.04 *μ*m/min for treated cells. Cells treated with AKTi XII exhibit a 24% reduction of mean velocity.

We calculated also the Mean Square Displacement (MSD) <r^2^(Δt)> as a function of time lag Δt (28). In Fig. 6B the logarithmic plot of MSD is shown. Here, both control and treated cells exhibit super-diffusive motion for small Δt (see for comparison the logarithmic slope of 2, which represents a quasi ballistic motion, and slope of 1, which corresponds to random walks in Brownian motion) and conversely, approach sub-diffusive motion for large time lags. Besides, for each time interval considered, untreated cells migrated further than cells treated with AKTi XII. Overall, the movement and directionality analyses show speed rate decrease and compromised status of the treated cell culture. An example of trajectories recovered from the sequence CTRL 3c and XII 3c is shown in Fig. 6C and D, respectively. These trajectories depict in an explicatory manner the role of inhibitor XII in cell motility. Control cell trajectories showed higher displacements than those revealed on the treated MSC. Fig. 6E shows three subsequent frames (acquired at time lapse Δt = 5 min) extracted from the same sequence showing tracked and labeled cells. While the movement of some cells is reduced (cell labels appear almost in the same position over this limited period of time), cells labeled 46 and 47 show a more dynamic interaction in this time frame. In general, considering the whole sequence, untreated cells tend to move faster than those treated with inhibitor XII.

## Discussion

MSC are released from the bone marrow and enter into the peripheral circulation where, in homeostatic conditions, they contribute to tissue repair. Moreover, inflammatory chemokines, cytokines and growth factors released upon damage provide migratory cues that drive their mobilization to tissue injury sites to participate in immune modulation, tissue remodeling and wound healing (29). Taking advantage of their homing capacities, MSC targeted migration has been used as delivery vehicle for treatments against cancer, graft versus host disease, arthritis, multiple sclerosis, and many other diseases. MSC migration toward the inflammatory signals produced by the wounded environment has been widely studied, nevertheless the true migratory mechanism has yet to be elucidated.

PI3K/AKT signaling regulates multiple biological processes such as cell division, apoptosis, cell growth and cell migration. A series of recent studies documents the isoform-specific functions of AKT family members 1 and 2 in regulation of cellular motility and migration by influencing numerous cellular targets involved in organization of the actin cytoskeleton, cellular interaction with the extracellular matrix, expression of motility genes and establishment of cellular polarity (30). In particular, AKT1 and AKT2 opposing functions on migration of breast cancer epithelial cell lines have been firmly established. AKT2 promotes mammary epithelial cell migration and invasion, as shown by siRNA-mediated depletion of AKT2, through upregulation of β1-integrin and increased stability of palladin expression (6,31). Conversely, AKT1 negatively affects cell migration by either transcriptional regulation of motility genes, or actin organization and formation of stress fibers and cellular interaction with the extracellular matrix (6,31). The role of AKT isoforms in other cell models is less clear. In particular, both AKT1 and 2 seem to play an inhibitory role in prostate cancer cell migration and invasion (32).

In this study, we examined human MSC migration and wound closure potential *in vitro*. We also used selective inhibitors of AKT isoforms 1 and 2 to dissect the contribution of each isoform to migration, by selectively blocking either isoform 2 only or both isoforms. Our results showed that both isoforms are expressed at high level in MSC obtained from three different individual and cultured *in vitro*. Reactivity to anti-pSer473 further demonstrates their activity. Of note, only selective inhibition of AKT2 effectively counteracted wound closure, whereas dual inhibition of AKT1 and 2 was less effective. An explanation for this result comes from previous reports, showing that AKT1 inhibition can lead to increased cell migration. Hence, in our cell system blocking both isoforms weakens the efficacy of AKT2 inhibition. To our knowledge, this is the first study that provides evidence for an isoform-specific positive role of AKT in regulating migration of human mesenchymal stem cells.

Although we have a deep understanding of the mechanisms by which the AKT kinase is regulated, what ultimately qualifies a kinase are its substrates, as it is very well known that the phosphorylation of specific substrates determines most of protein kinase cellular functions (33). Recently, the mechano-sensor and actin bundling protein Ankrd2 was demonstrated to be a specific AKT2 substrate (14). It is therefore tempting to speculate that AKT2 might promote MSC migration through direct phosphorylation of Ankrd2. However, the relative contribution, if any, of Ankrd2 to the phenotypic changes associated with activation of AKT2 needs deeper investigation.

MSC are used with increasing frequency in laboratories and clinics as potential treatments for injury and tissue regeneration. The fundamental mechanisms through which MSC migrate to sites of injury remains undefined. Our finding that AKT2 is required for directed chemotaxis by MSC offers a potential target of clinical relevance for enhancing or blocking the effects of migrating MSC.

## Figures and Tables

**Table I.** FACS analysis of cell cycle and apoptosis.^a^

	CTRL (%)	VIII (%)	XII (%)
G0/G1 phase			
0 h	90	94	94
24 h	93	90	87
S phase			
0 h	0.7	0.8	0.7
24 h	0.1	0.9	0.7
G2/M phase			
0 h	1.3	2.3	2.5
24 h	3.6	1.4	2.1
h-apoptosis			
0 h	6.5	2.1	1.6
24 h	1.4	6.6	7.3

a For detection and quantification of cell cycle distribution, samples containing 5×10^5^ cells were harvested by centrifugation, fixed in cold ethanol and subjected to propidium iodide (PI) staining.

## References

[1] Charbord P (2010). Bone marrow mesenchymal stem cells: historical overview and concepts. Hum Gene Ther.

[2] Devine SM, Cobbs C, Jennings M, Bartholomew A, Hoffman R (2010). Mesenchymal stem cells distribute to a wide range of tissues following systemic infusion into nonhuman primates. Blood.

[3] Chamberlain G, Fox J, Ashton B, Middleton J (2007). Concise review: mesenchymal stem cells: their phenotype, differentiation capacity, immunological features, and potential for homing. Stem Cells.

[4] Kang SK, Shin IS, Ko MS, Jo JY, Ra JC (2012). Journey of mesenchymal stem cells for homing: strategies to enhance efficacy and safety of stem cell therapy. Stem Cells Int.

[5] Manning BD, Cantley LC (2007). AKT/PKB signaling: navigating downstream. Cell.

[6] Chin YR, Toker A (2011). Akt isoform-specific signaling in breast cancer: uncovering an anti-migratory role for palladin. Cell Adh Migr.

[7] Worster DT, Schmelzle T, Solimini NL, Lightcap ES, Millard B, Mills GB, Brugge JS, Albeck JG (2012). Akt and ERK control the proliferative response of mammary epithelial cells to the growth factors IGF-1 and EGF through the cell cycle inhibitor p57Kip2. Sci Signal.

[8] Kakinuma N, Roy BC, Zhu Y, Wang Y, Kiyama R (2008). Kank regulates RhoA-dependent formation of actin stress fibers and cell migration via 14-3-3 in PI3K-Akt signaling. J Cell Biol.

[9] Cenni V, Sirri A, Riccio M, Lattanzi G, Santi S, de Pol A, Maraldi NM, Marmiroli S (2003). Targeting of the Akt/PKB kinase to the actin skeleton. Cell Mol Life Sci.

[10] Scita G, Tenca P, Frittoli E, Tocchetti A, Innocenti M, Giardina G, Di Fiore PP (2000). Signaling from Ras to Rac and beyond: not just a matter of GEFs. EMBO J.

[11] Qian Y, Corum L, Meng Q, Blenis J, Zheng JZ, Shi X, Flynn DC, Jiang BH (2004). PI3K induced actin filament remodeling through Akt and p70S6K1: implication of essential role in cell migration. Am J Physiol Cell Physiol.

[12] Enomoto A, Murakami H, Asai N, Morone N, Watanabe T, Kawai K, Murakumo Y, Usukura J, Kaibuchi K, Takahashi M (2005). Akt/PKB regulates actin organization and cell motility via Girdin/APE. Dev Cell.

[13] Yoeli-Lerner M, Yiu GK, Rabinovitz I, Erhardt P, Jauliac S, Toker A (2005). Akt blocks breast cancer cell motility and invasion through the transcription factor NFAT. Mol Cell.

[14] Cenni V, Bavelloni A, Beretti F, Tagliavini F, Manzoli L, Lattanzi G, Maraldi NM, Cocco L, Marmiroli S (2011). Ankrd2/ARPP is a novel Akt2 specific substrate and regulates myogenic differentiation upon cellular exposure to H(2)O(2). Mol Biol Cell.

[15] Irie HY, Pearline RV, Grueneberg D, Hsia M, Ravichandran P, Kothari N, Natesan S, Brugge JS (2005). Distinct roles of Akt1 and Akt2 in regulating cell migration and epithelial-mesenchymal transition. J Cell Biol.

[16] Pierini M, Dozza B, Lucarelli E, Tazzari PL, Ricci F, Remondini D, di Bella C, Giannini S, Donati D (2012). Efficient isolation and enrichment of mesenchymal stem cells from bone marrow. Cytotherapy.

[17] Maraldi T, Riccio M, Resca E, Pisciotta A, La Sala GB, Ferrari A, Bruzzesi G, Motta A, Migliaresi C, Marzona L, De Pol A (2011). Human amniotic fluid stem cells seeded in fibroin scaffold produce in vivo mineralized matrix. Tissue Eng Part A.

[18] Dozza B, Gobbi G, Lucarelli E, Pierini M, Bella CD, Frisoni T, Tazzari PL, Ricci F, Mirandola P, Carubbi C, Giannini S, Donati D, Vitale M (2012). A rapid method for obtaining mesenchymal stem cells and platelets from bone marrow aspirate. J Tissue Eng Regen Med.

[19] Maraldi T, Riccio M, Sena P, Marzona L, Nicoli A, La Marca A, Marmiroli S, Bertacchini J, La Sala G, De Pol A (2009). MATER protein as substrate of PKCepsilon in human cumulus cells. Mol Hum Reprod.

[20] Cenni V, Sabatelli P, Mattioli E, Marmiroli S, Capanni C, Ognibene A, Squarzoni S, Maraldi NM, Bonne G, Columbaro M, Merlini L, Lattanzi G (2005). Lamin A N-terminal phosphorylation is associated with myoblast activation: impairment in Emery-Dreifuss muscular dystrophy. J Med Genet.

[21] Duca M, Dozza B, Lucarelli E, Santi S, Di Giorgio A, Barbarella G (2010). Fluorescent labeling of human mesenchymal stem cells by thiophene fluorophores conjugated to a lipophilic carrier. Chem Commun (Camb).

[22] Maraldi T, Bertacchini J, Benincasa M, Guida M, De Pol A, Liotta LA, Petricoin E, Cocco L, Marmiroli S (2011). Reverse-phase protein microarrays (RPPA) as a diagnostic and therapeutic guide in multidrug resistant leukemia. Int J Oncol.

[23] Gebäck T, Schulz MM, Koumoutsakos P, Detmar M (2009). TScratch: a novel and simple software tool for automated analysis of monolayer wound healing assays. Biotechniques.

[24] Mirandola P, Sponzilli I, Gobbi G, Marmiroli S, Rinaldi L, Binazzi R, Piccari GG, Ramazzotti G, Gaboardi GC, Cocco L, Vitale M (2006). Anticancer agents sensitize osteosarcoma cells to TNF-related apoptosis-inducing ligand downmodulating IAP family proteins. Int J Oncol.

[25] Martelli AM, Evangelisti C, Follo MY, Ramazzotti G, Fini M, Giardino R, Manzoli L, McCubrey JA, Cocco L (2011). Targeting the phosphatidylinositol 3-kinase/Akt/mammalian target of rapamycin signaling network in cancer stem cells. Curr Med Chem.

[26] Wu W-I, Voegtli WC, Sturgis HL, Dizon FP, Vigers GPA, Brandhuber BJ (2010). Crystal structure of human AKT1 with an allosteric inhibitor reveals a new mode of kinase inhibition. PLoS One.

[27] Chin YR, Toker A (2010). The actin-bundling protein palladin is an Akt1-specific substrate that regulates breast cancer cell migration. Mol Cell.

[28] Nocentini S, Reginensi D, Garcia S, Carulla P, Moreno-Flores MT, Wandosell F, Trepat X, Bribian A, Del Río JA (2012). Myelin-associated proteins block the migration of olfactory ensheathing cells: an in vitro study using single-cell tracking and traction force microscopy. Cell Mol Life Sci.

[29] Fox JM, Chamberlain G, Ashton BA, Middleton J (2007). Recent advances into the understanding of mesenchymal stem cell trafficking. Br J Haematol.

[30] Stambolic V, Woodgett JR (2006). Functional distinctions of protein kinase B/AKT isoforms defined by their influence on cell migration. Trends Cell Biol.

[31] Toker A (2012). Achieving specificity in Akt signaling in cancer. Adv Enzyme Regul.

[32] Virtakoivu R, Pellinen T, Rantala JK, Perälä M, Ivaska J (2012). Distinct roles of AKT isoforms in regulating β1-integrin activity, migration, and invasion in prostate cancer. Mol Biol Cell.

[33] Moritz A, Li Y, Guo A, Villén J, Wang Y, MacNeill J, Kornhauser J, Sprott K, Zhou J, Possemato A, Ren JM, Hornbeck P, Cantley LC, Gygi SP, Rush J, Comb MJ (2010). AKT-RSK-S6 kinase signaling networks activated by oncogenic receptor tyrosine kinases. Sci Signal.

